# Supplementary material for: Species-Specific Humoral Immune Responses in Sheep and Goats upon Small Ruminant Lentivirus Infections Inversely Correlate with Protection against Virus Replication and Pathological Lesions
Source: Int J Mol Sci. 2021 Sep 11;22(18):9824. doi: 10.3390/ijms22189824 (PMC8467527; doi:10.3390/ijms22189824)
Supplement: Supplementary file 1 [file ijms-22-09824-s001.zip › ijms-1349512-supplementary.pdf]

Supplementary Figure S1: Antibody response of sheep and goats infected with a genotype A and B strains over a 9 month's time period. Animals infected with the genotype A and B strains were inoculated at one week interval and blood samples were collected every two weeks until 4 months p.i and then every month until the end of the experiment. The dotted line represents the cut-off value of the test which considered any S/P values above 60% as positive.

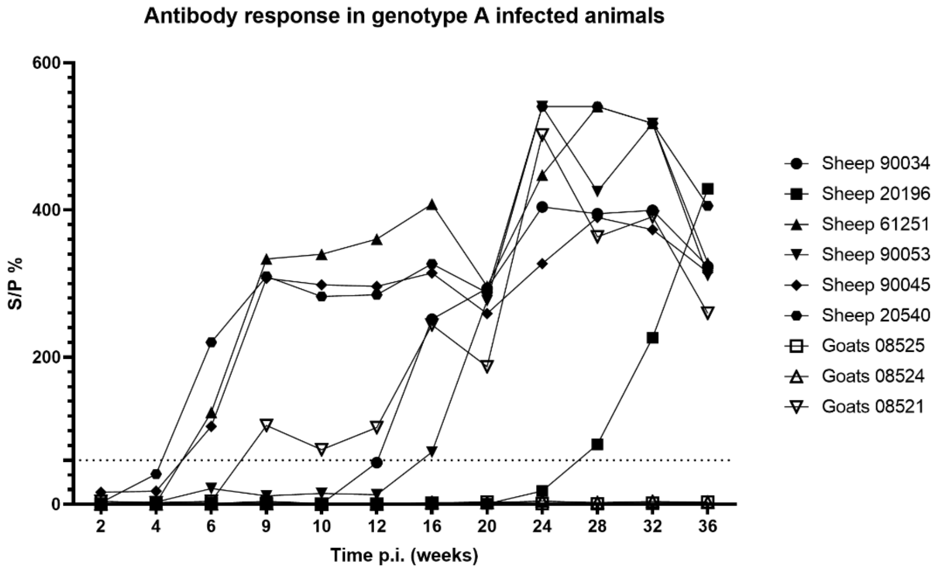

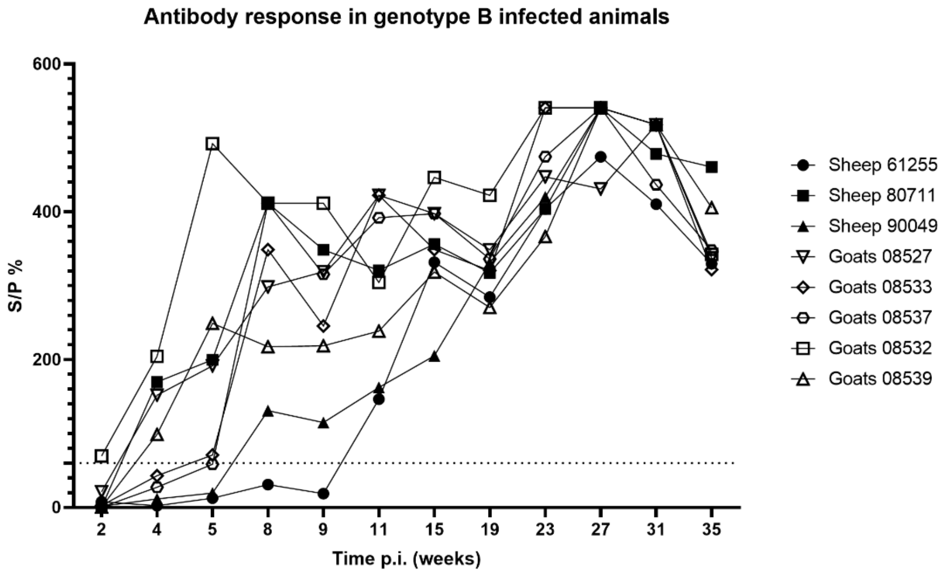

Supplementary Figure S2: Overview of the experimental infection set-up on sheep and goats infected with a genotype A and B SRLV strains. At day 0, 6 sheep and 3 goats were intratracheally inoculated with 1 ml of the genotype A strain (dose: $10^{2.66}$ $TCID_{50}$/mL) and 3 sheep and 6 goats with 1ml of the genotype B strain (dose: $10^{4.66}$ $TCID_{50}$/mL). At 4 weeks post infection, groups of 3 inoculated sheep or goats with the genotype A or B strain, respectively, were moved to separate pens and 3 contact sheep or goats were added to evaluate potential intra- and cross-species transmissions. Animals were distributed over 7 pens with a density of 6 animals per pens. Only 5 animals instead of 6 were housed in the control pen. Unexpectedly, one goat and one sheep infected with the genotype B strain died at week 2 and week 32 respectively. The experiment lasted for 9 months.

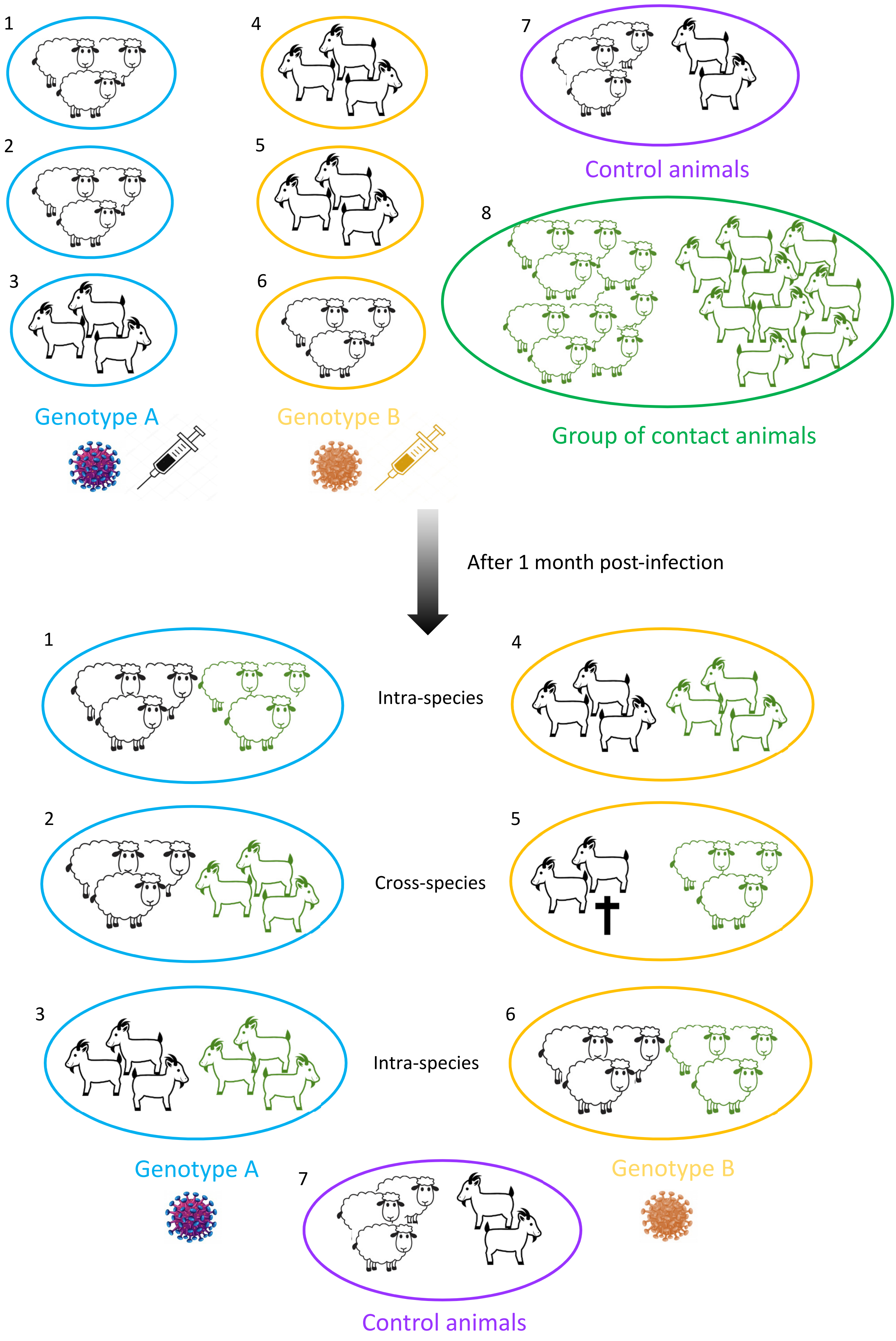
1
2
3
4
5
6
7
8
Genotype A
Genotype B
Control animals
Group of contact animals
After 1 month post-infection
1
4
Intra-species
2
5
Cross-species
3
6
Intra-species
Genotype A
Genotype B
7
Control animals
